# TP53 Deficiency in the Natural History of Prostate Cancer

**DOI:** 10.3390/cancers17040645

**Published:** 2025-02-14

**Authors:** Heidemarie Ofner, Gero Kramer, Shahrokh F. Shariat, Melanie R. Hassler

**Affiliations:** 1 Department of Urology, Medical University of Vienna, 1090 Vienna, Austria; heidemarie.ofner@meduniwien.ac.at (H.O.); gero.kramer@meduniwien.ac.at (G.K.); shahrokh.shariat@gmail.com (S.F.S.); 2 Hourani Center for Applied Scientific Research, Al-Ahliyya Amman University, Amman 19328, Jordan; 3 Department of Urology, University of Texas Southwestern Medical Center, Dallas, TX 75390, USA; 4 Department of Urology, Second Faculty of Medicine, Charles University, 150 06 Prague, Czech Republic; 5 Department of Urology, Weill Cornell Medical College, New York, NY 10065, USA; 6 Karl Landsteiner Institute of Urology and Andrology, 1090 Vienna, Austria

**Keywords:** prostate cancer (PCa), metastatic hormone-sensitive prostate cancer (mHSPC), castration-resistant prostate cancer (CRPC), TP53 alteration, genetics

## Abstract

Prostate cancer remains a leading cause of cancer-related mortality in men, with advanced stages posing significant treatment challenges due to high morbidity and mortality. Among genetic alterations, TP53 mutations are among the most prevalent in cancers and are strongly associated with poor clinical outcomes and therapeutic resistance. This review investigates the role of TP53 mutations in prostate cancer progression, prognosis, and therapeutic development. A comprehensive analysis of preclinical and clinical studies was conducted to elucidate the molecular mechanisms, clinical implications, and potential therapeutic approaches associated with TP53 alterations in prostate cancer. TP53 mutations are highly prevalent in advanced stages, contributing to genomic instability, aggressive tumor phenotypes, and resistance to standard treatments. Emerging evidence supports the utility of liquid biopsy techniques, such as circulating tumor DNA analysis, for detecting TP53 mutations, providing prognostic value and facilitating early intervention strategies. Novel therapeutic approaches targeting TP53 have shown promise in preclinical settings, but their clinical efficacy requires further validation. Overall, TP53 mutations represent a critical biomarker for disease progression and therapeutic response in prostate cancer. Advances in detection methods and targeted therapies hold significant potential to improve outcomes for patients with TP53-mutated prostate cancer. Further research is essential to integrate TP53-based strategies into routine clinical practice.

## Simple Summary

Prostate cancer, one of the most prevalent malignancies in men worldwide, is associated with significant morbidity and mortality. Among the genetic alterations driving prostate cancer progression, mutations in TP53 play a pivotal role in tumor resistance to therapeutic interventions and the promotion of metastasis. This review examines the critical role of TP53 mutations in prostate cancer, emphasizing their contribution to disease progression and their influence on clinical outcomes. By synthesizing the current literature, we aim to underscore how a deeper understanding of the molecular underpinnings of prostate cancer, particularly its mutational landscape, can inform and enhance personalized treatment strategies. Furthermore, the review discusses emerging therapeutic approaches targeting TP53, offering potential avenues for novel treatments in patients with advanced disease.

## 1. Introduction

An estimated 1.4 million cases of prostate cancer (PCa) were diagnosed in 2020 and an estimated 375,000 men died from the disease in the same year, making it the seventh most common cause of cancer-related deaths among males globally [1]. Over the past 20 years, the incidence and prevalence of metastatic PCa have risen across all age groups, and the development of a metastatic castrate-resistant state is linked to PCa’s lethality. Since this surge in cases will not be preventable by public health or lifestyle interventions, gaining a better understanding of the cancer biology and natural history of the disease remains a crucial burden. Even though the presence of a distant metastatic disease at the time of diagnosis is rare, approximately 20–40% of patients suffer from a relapse of disease with a biochemical recurrence after primary local treatment [2]. PCa is associated with significant clinical heterogeneity, with many tumors showing slow evolutionary trajectories and a smaller group with aggressive metastatic behavior that leads to premature death. Treatment strategies for advanced PCa follow the goal to prolong survival through targeting key drivers of carcinogenesis, where the androgen receptor pathway plays a crucial role and androgen deprivation therapy (ADT) remains the backbone of therapy in metastatic hormone-sensitive prostate cancer (mHSPC) in combination with other novel agents [3]. Despite major therapeutic advances in recent years, mHSPC remains incurable and patients become castration-resistant (mCRPC); a state of disease with high morbidity and mortality. The heterogeneity of PCa is based on different molecular changes that involve genetic alterations, gene expression signatures, and signaling pathways with distinct impacts on PCa aggressiveness. A better understanding of these molecular changes and PCa’s molecular signatures has led to the development of new diagnostic tests and targeted therapies for PCa patients. For example, a major advancement in PCa treatment in the last few years was the finding that a significant number of PCa patients harbor somatic or germline mutations in homologous recombination deficiency (HRD) genes such as *BRCA1* or *BRCA2*, and targeting these alterations via PARP-inhibitors has been successfully tested in clinical trials, leading to FDA and EMA approval for PARP-inhibitors in mCRPC patients [4,5,6]. However, other frequent alterations, such as *TP53* mutations, are also associated with a significantly worse prognosis, and to date, no specific therapeutic approach is recommended for these patients [7]. *TP53* mutations can be detected in up to 30% of localized and 40–50% of metastatic PCa, making *TP53* gene alterations a common somatic event in advanced PCa [8,9]. After the AR gene, it is the gene most commonly disrupted in castration-resistant PCa. In certain situations, chromothripsis—a complex, catastrophic disorder of the genome—can result from *TP53* inactivation, which causes genomic instability with inverted rearrangements. Gene mutations and significant deletions affecting the *TP53* locus on the 17p chromosome are examples of somatic *TP53* changes. They have particular effects on the immune system and the therapeutic efficacy of medication classes, counteract the impact of AR inhibitors, and increase the rate at which PCa cells proliferate [10,11].

In this review, we aim to give an overview of *TP53’s* cellular function and discuss available evidence on the role of *TP53* mutations in prostate cancer regarding its impact on clinical outcomes throughout the disease.

## 2. *TP53*—The Guardian of the Genome in Malignant Diseases

The tumor suppressor protein p53 is an important transcription factor that plays a pivotal role in maintaining cellular homeostasis by regulating a variety of genes and cellular processes. Because of its wide-ranging functions in cellular regulation, p53 has been identified as a driver mutation for numerous malignancies including PCa and is implicated in almost all known cancer hallmarks [12]. Regarding its function and regulation in the cell, p53 activity is low under non-stress conditions due to suppression by the proteins MDM2 and MDMX, which target p53 for degradation [13]. However, stress factors such as DNA damage or oncogene activation release p53 from these suppressors, allowing it to enter the nucleus, where it promotes cell cycle arrest, apoptosis, and DNA repair via its role as a transcription factor—key mechanisms that prevent cancerous growth. Beyond these primary functions, p53 also contributes to anticancer defense by regulating antioxidant responses, metabolism, and immune function, among others [14,15].

The action of p53 varies by tissue type, with gene expression profiles differing significantly across organs. For example, after exposure to ionizing radiation, gastrointestinal and lymphoid tissues exhibit distinct p53 activation patterns, which in turn influence their radiation sensitivities [16]. For PCa, it has been reported that cell lines with defective *TP53* are less sensitive to radiotherapy [17]. The cellular response to p53 is also temporally regulated: transient p53 activation often leads to repair and survival, whereas prolonged activation induces apoptosis or cellular senescence [18]. Studies in mice with genetic deletions of p53 reveal the high incidence of cancer in the absence of this protein, reinforcing p53’s role as a fundamental tumor suppressor [19]. For example, in mice with prostate-specific deletion of both *PTEN* and *TP53*, prostate tumors develop much earlier than in *PTEN* knock-outs, which is due to the absence of *TP53*-induced cellular senescence [20]. In humans, germline *TP53* mutations cause Li–Fraumeni syndrome, a condition marked by early-onset cancers across multiple tissue types [21]. Genomic analyses in human cancers further showed that *TP53* mutations belong to the most common alterations across all cancer types, with mCRPC harboring alterations in up to 50% of samples tested and contributing to chromosomal instability, oncogene amplification, and poor prognosis in patients with mutated p53 profiles [7].

**Figure 1:**
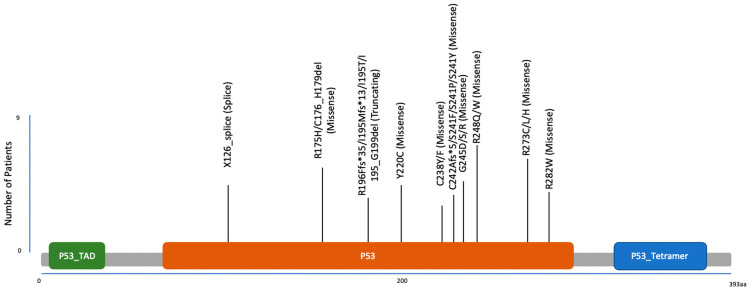
Mutation diagram of localization and frequency of splice, missense, and truncating tp53 mutations in a cohort of 429 metastatic PCa patients [34].

**Figure 2:**
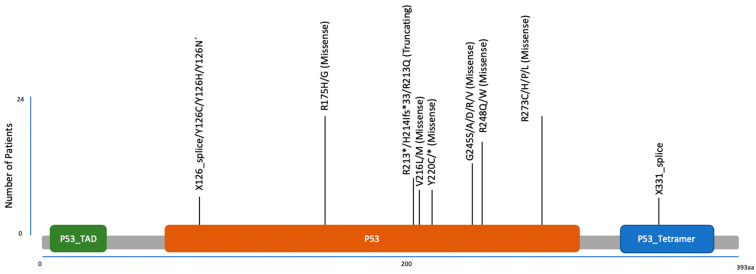
Mutation diagram of localization and frequency of splice, missense, and truncating tp53 mutations in a cohort of 1465 localized PCa patients [35].

Mutations in *TP53* predominantly occur in its DNA-binding domain, disrupting its ability as a transcription factor to bind and regulate target genes effectively [22]. These mutations are often missense, with certain “hotspot” residues recurring frequently across different cancer types. For example, in mCRPC, but also in localized PCa, one of the most frequent *TP53* mutations is the R248Q mutation in the DNA binding domain [8] (Figure 1 and Figure 2). Functionally, the R248Q mutant may exhibit a gain-of-function (GOF) effect by interfering with and suppressing wild-type (WT) p53 activity, thereby contributing to tumor progression and metastasis, making it even more oncogenic than a complete loss of p53 [23]. GOF mutations lead to enhanced cell proliferation, migration, and genomic instability, as well as interactions with the tumor microenvironment (TME) that promote malignancy. Importantly, cancer cells often become “addicted” to mutant p53, as its depletion in these cells reduces their malignant traits, making mutant p53 a viable therapeutic target [24]. TP53 loss or mutation also enables tumor cells to evade both adaptive and innate immune systems, and re-shapes the TME. For example, it has been shown that tumor cells with defective p53 exhibit reduced MHC class I and II expression [25,26]. Consequently, these cells become less recognizable to cytotoxic T lymphocytes. Additionally, wild-type p53 inhibits immune checkpoint molecules like PD-L1, while p53 mutations increase PD-L1 expression, promoting immune evasion [27]. Mutant p53 also alters the tumor secretome to suppress T cell activity [28]. These alterations result in an immune-suppressive microenvironment and diminished T cell responses. Furthermore, innate immune cells, such as natural killer (NK) cells and macrophages, can also be inhibited by p53 mutations [29,30,31]. WT p53 upregulates ligands that enhance NK cells’ ability to recognize and kill MHC-defective tumor cells, while mutant p53 decreases their expression, enabling immune escape [29]. Moreover, p53-null tumor cells show resistance to apoptosis, further impairing NK cell cytotoxicity [31]. Regarding macrophages, p53-null cancer cells have been reported to resist macrophage-mediated phagocytosis by producing extracellular vesicles enriched with immunosuppressive PD-L1 [30], and the tumor microenvironment (TME) shaped by p53-mutant cells fosters immune evasion through myeloid cell reprogramming and regulatory T cell (Treg) recruitment [32,33].

In summary, p53’s complex role in cancer suppression stems from its ability to regulate diverse cellular functions and respond to stress stimuli, with tissue-specific and mutation-specific effects on tumor biology. Targeting p53 dysfunction in PCa holds potential for new treatment modalities aimed at leveraging the protein’s central role in cell regulation and tumor suppression.

## 3. *TP53* and Prostate Cancer

### 3.1. TP53 Alterations and Preclinical Data in PCa Models

Several preclinical studies have investigated the role of TP53 in the progression of PCa, in combination with other mutations or regarding therapy responses in in vitro and in vivo PCa models. Some of these studies confirmed mechanistic data on TP53 regulation reported in other cancer types and provided preclinical biological insight into correlations found in clinical samples. For example, the inhibitory function of MDM2 and MDMX was tested in an androgen-sensitive PCa cell line carrying WT TP53 and showed that, when MDM2 and MDMX were inhibited, the TP53 pathway was activated and AR levels and function decreased [36].

In clinical PCa samples, an increase in the frequency of TP53 alterations is found along the course of the disease, i.e., from localized disease to mCRPC samples. In vitro data investigating androgen-sensitive LNCap cells with and without TP53 alterations that were co-cultured showed that the subpopulation carrying the TP53 alteration became the dominant subpopulation after several days in culture [37]. This growth advantage was observed in hormone-sensitive and, to a larger extent, castration conditions, which is in line with the finding of a longitudinal increase in TP53 alterations during disease progression. The TP53-altered subpopulation was characterized by low CDKN1A expression (a tumor suppressor and target of functional p53), indicating that intact p53 served as a barrier to cell proliferation and advancement to castration-resistant growth [37]. Furthermore, TP53-deficient cells harbored more copy number variants (CNVs) when exposed to cytotoxic compounds stimulating CNV occurrence and also demonstrated a survival advantage in response to a CNV-inducing agent [37]. Thus, the loss of TP53 contributed to the development of CRPC by promoting the proliferation and genomic instability of tumor cells. Co-occurrence of alterations in TP53 and other important tumor suppressors, in particular RB1 or PTEN, was shown to render PCa tumor cells more aggressive or more resistant towards conventional therapies. For example, in PCa cell lines, RB1 loss enhanced ionizing radiation-induced DNA damage and promoted cellular senescence through a TP53-dependent pathway, but double deletion of RB1 and TP53 reversed DNA damage-induced cellular senescence and promoted radiation survival, although radiosensitivity could be restored by PARP1 inhibitor treatment [38]. Early in vivo studies in PTEN knock-out mice, which develop PCa after long latency due to the activity of the TP53-dependent cellular senescence pathway, showed that combined PTEN and TP53 inactivation elicits PCa in young mice and leads to early lethality in the absence of TP53 dependent cellular senescence [20].

However, different combinations of tumor suppressor and oncogenic alterations may have divergent effects on PCa proliferation and development. For example, in a mouse model with PTEN/TP53 alterations, functional RB1, and overexpression of the ERG oncogene, ERG overexpression blocked the PTEN/TP53-dependent decrease in AR expression, the expression of cell cycle-related genes, and the expression of mesenchymal lineage regulators, thus restricting lineage plasticity and maintaining androgen sensitivity [39]. Interestingly, co-occurrence of the TPMRSS2-ERG fusion and a gain-of-function TP53 mutant was shown to accelerate PCa growth in vitro and in mice by activating beta-catenin expression and promoting pyrimidine synthesis, indicating that different TP53 mutations or deletions may have distinct effects during PCa progression [23]. This context-dependent phenotype of TP53 alterations was also investigated in a study on specific TP53 mutants (R273C and R273H) in a WT TP53-null setting and in the presence of endogenous WT TP53, revealing that the mutants led to pro-tumorigenic transcriptional activity, but only in the presence of WT TP53 [40].

Several in vitro studies have tested the effects of systemic therapies on PCa proliferation in the context of TP53 alterations. For example, deletion of the DNA repair gene MMS22L was found to make PCa cells hypersensitive to PARP inhibitors in a CRISPR knock-out screen; however, this effect was only seen when p53 was still intact [11]. Another study investigated the effects of GnRH agonists in PCa cell lines with and without TP53. It showed that GnRH agonists increase the expression of proapoptotic proteins through phosphorylation/activation of Ser-15 of TP53, triggered by p38 MAPK phosphorylation. GnRH agonists also sensitized and re-sensitized docetaxel-resistant PCa cells to docetaxel, but failed to do so if TP53 was absent, indicating that a functional p53 protein was necessary in combination treatment of GnRH agonists and docetaxel re-challenge [41].

Recent therapies for advanced PCa usually combine systemic GnRH agonists or antagonists with androgen-receptor targeting agents (ARTAs), such as abiraterone or enzalutamide. After progression on ARTAs, approximately 20% of tumors show a neuroendocrine lineage transformation, rendering them more resistant to ADT and successive therapies [8,42]. To gain mechanistic insights and identify key drivers of this transition, in vitro PCa models were used that were treated with ARTAs until resistance occurred. Loss of TP53 and RB1 was shown to induce a shift from androgen-dependent luminal cells to androgen-independent basal cells mediated by the reprograming transcription factor SOX2, thus contributing to lineage plasticity promoting resistance through lineage switching [43]. Something that is noteworthy is the fact that not all cells with RB1/TP53 defects in this study underwent lineage plasticity and the AR program remained active, however, these cells were associated with a stem-cell-like program, the induction of lineage plasticity genes, and the shorter duration of response to androgen-deprivation or ARTA therapy.

Regarding the role of the immune system in PCa development, *TP53* loss was associated with the induction of a pro-inflammatory and immunosuppressive TME in a murine PCa model [44]. Another study reported that infiltrating mast cells may lead to reduced PCa chemotherapy and radiotherapy sensitivity via activation of p38/p53/p21 signaling in in vivo mouse models [45]. Furthermore, the immune checkpoint inhibitor B7-H3 (encoded by CD276) involved in immune suppression has been reported to be elevated in PCa tumors with *PTEN* and *TP53* defects via activation of the transcription factor SP1. A preclinical model of PTEN/p53-deficient mice showed immunological escape mechanisms that take place when the immune checkpoint B7-H3 associated with *TP53* deletions is overexpressed. Elevated B7-H3 induced tumor growth and contributed to the immunosuppression of tumor-killing T cells and NK cells in *PTEN/TP53*-deficient tumors [10]. Targeting B7-H3 together with PD-L1/CTLA4 checkpoint inhibitors showed curative potential in *PTEN/TP53*-deficient CRPC models.

### 3.2. TP53 Alterations and Clinical Prognosis in PCa Patients

#### 3.2.1. Localized Prostate Cancer and Biochemically Recurrent Prostate Cancer

**Figure 3:**
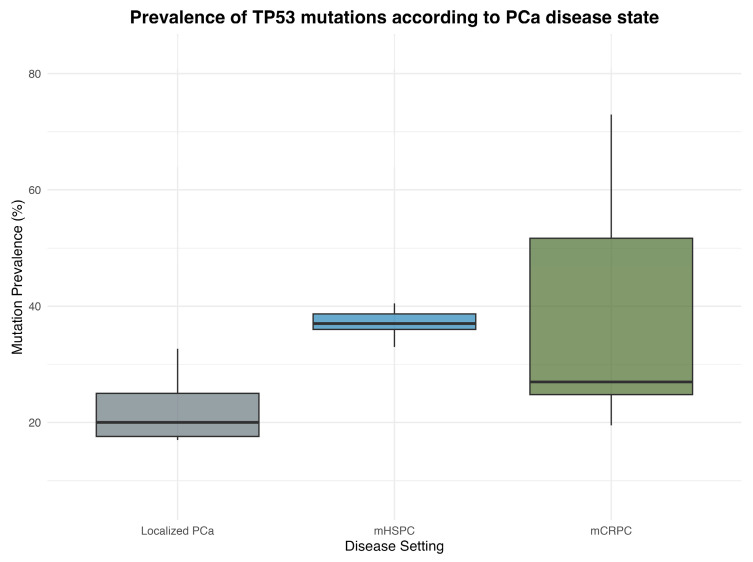
Prevalence of TP53 mutations in localized PCa, mHSPC, and mCRPC according to cited published data [46,47,48,52,53,54,55,56,57,58,59,60,61].

In localized PCa with a primary Gleason 5 pattern, 33% of patient tumors showed *TP53* mutations [46]. The presence of *TP53* in localized PCa was associated with biochemical recurrence (BCR), metastasis, and worse overall survival in univariable analysis [46,47]. Genomic profiling of lethal primary PCa tumors reported *TP53* alterations in 27% of samples, whereas in RPE samples from intermediate- or high-risk disease *TP53* mutations were present in 18% [47,48] (Figure 3). In the former study, *TP53* alterations were not associated with time to CRPC but when comparing same-patient primary treatment-naïve and mCRPC samples, an increase in *TP53* alterations in mCRPC was reported [47]. The latter study reported a higher risk of PSA persistence/recurrence in patients with somatic *TP53* alterations, which was also found in other studies [48,49]. According to profiling of matched tumors from individual patients, somatic *TP53* alterations appeared early in localized tumors from patients who later had metastatic illness, indicating that the presence of *TP53* alterations may predict the increased risk of progression to metastatic disease [50]. In line with this, another study reported the presence of *TP53* mutations at low frequency in primary samples and enrichment of *TP53* mutations in matched longitudinal metastatic samples [51]. Alterations in *TP53* in localized or metastatic hormone-sensitive PCa also had a shorter time to CRPC, and cumulative gene hits in *TP53*, *PTEN*, and *RB1* led to an incremental risk of progression with inferior OS with increasing gene hits [52].

#### 3.2.2. Metastatic Hormone-Sensitive Prostate Cancer

In metastatic PCa, the presence of combined alterations in *TP53*, *PTEN*, and *RB1* has been linked to poorer OS, an increasing prevalence of *ETS* gene fusions, and gene expression patterns favoring aggressive disease and tumor progression [62]. *TP53* and *SPOP* mutations were found to be mutually exclusive, with *TP53* alterations as negative and *SPOP* mutations as positive prognostic markers in metastatic PCa [53]. Enrichment of *TP53* alterations was also detected in tumors from patients with primary PCa and wide-spread metastasis compared to those without (40% vs. 20%), and *TP53* alterations were associated with shorter OS [63]. When specifically comparing secondary to primary mHSPC, *TP53* mutations seemed to be associated with poor prognosis in secondary but not primary mHSPC [64].

A gene expression score based on *TP53*, *PTEN,* and *RB1* expression showed that low expression of these three genes was correlated with lower CRPC-free survival and OS [65]. mHSPC patients without mutations in these genes derived a benefit from ADT + docetaxel treatment but not from ADT treatment alone [66].

In mHSPC, current guidelines recommend treatment strategies based on tumor volume, with higher tumor volumes requiring a more aggressive approach [3]. However, the impact of *TP53* status on tumor volume and prognosis is unclear. Interestingly, in a subset of de-novo mHSPC samples from the STAMPEDE trial, *TP53* alterations were more frequent in low-volume compared to high-volume disease [54]. In mHSPC men with oligometastasis, *TP53* driver mutations were associated with shorter radiographic progression-free survival (rPFS) and time to CRPC, whereas mHSPC with high volume but without *TP53* alterations had a better rPFS than those with *TP53* alterations and similar to those with oligometastatic disease [67]. Metachronous oligometastatic mHSPC patients showed a lower long-term control rate if a *TP53* mutation was present (27.6% vs. 42.3%), and bone failure was more common with tumors with *TP53* mutations (44.8% vs. 25.9%) [68]. From these data, it can be hypothesized that the presence of a *TP53* alteration in oligometastatic or low-volume mHSPC is associated with a more aggressive disease course. Indeed, in biopsy samples from PCa patients, mutations in *TP53* were associated with visceral dissemination such as liver metastasis and early death, indicating a group of PCa patients that have a high risk of life-threatening disease who might benefit from more intensified treatment therapies [55]. *TP53* mutation prevalence in mHSPC in shown in Figure 3.

#### 3.2.3. Castration-Resistant Prostate Cancer

Early whole-exome and -transcriptome sequencing studies in mCRPC patients have shown that somatic *TP53* mutations are the most selectively mutated genes in mCRPC compared to primary PCa samples and, besides alterations in *AR*, the most frequent alteration found (in up to 50% of mCRPC samples) [7,69] (Figure 3). Regarding genomic structural variants such as deletions, insertions, duplications, inversions, and translocations, it was shown that biallelic *TP53* inactivation in mCRPC samples was significantly associated with the presence of chromothripsis, which occurred in a stochastic manner [69]. In an integrative analysis addressing genomic features and clinical outcomes of >400 mCRPC cases, alterations in *TP53* were associated with a shorter time on androgen receptor signaling inhibitor (ARSI) therapy, but there was no association with OS [8].

For mCRPC patients with features of neuroendocrine dedifferentiation, the presence of *TP53*, *RB1*, or *PTEN* alterations was associated with a longer PFS under chemotherapy than ARTA, with platinum-based chemotherapy showing longer median PFS and OS than docetaxel only [70].

Trials reporting the impact of TP53 mutations on prognosis in different disease stages are shown in Table 1. 

### 3.3. TP53 Alterations and Association with Therapy Outcomes

Regarding the predictive potential of TP53 for therapy outcomes, it has to be considered that TP53 status is a prognostic biomarker for PCa in various clinical scenarios, and pathogenic alterations are usually associated with worse prognosis. Thus, given its association with prognosis, it may be mistakenly assumed to be predictive when therapy outcomes are considered. Still, several studies have investigated the association between TP53 alteration status and treatment results. For example, in patients with high-risk localized PCa receiving anti-androgen therapies before prostatectomy, whole-exome and -transcriptome sequencing of pretreatment tumors showed that TP53 alterations were exclusively found in non-responders to neoadjuvant therapy [71].

For mHSPC patients, a study reported that patients receiving ADT + abiraterone, but not ADT + docetaxel, had a lower PFS if alterations in tumor suppressor genes such as TP53, PTEN, or RB1 were detected in pretreated tumors [56].

In oligometastatic mHSPC patients from the STOMP and ORIOLE trials, it was shown that metastasis-directed therapy (MDT) prolonged PFS compared to observation and that patients with a high-risk mutation in TP53 (or ATM, BRCA1/2, RB1) gained the greatest benefit from MDT (PFS of 7.5 months (95% CI, 5.9—not reached) with MDT compared to 2.8 months (95% CI, 2—not reached) without) [72].

For mCRPC patients, several studies have investigated outcomes for ARTA therapies when TP53 alterations are detected. The presence of TP53 alterations in the primary tumor was associated with shorter PFS under abiraterone or enzalutamide therapy [57]. Detection of TP53 alterations in circulating tumor DNA prior to ARTA start in mCRPC patients was also correlated with the development of rapid resistance and adverse prognosis [58,73], and a retrospective analysis of circulating tumor cells (CTCs) in CRPC patients after progression on abiraterone/enzalutamide showed that CTCs harboring TP53 mutations were enriched in resistant patients [74]. When analyzing plasma tumor DNA before and after one cycle of abiraterone, chemotherapy-naïve mCRPC patients who had an alteration in TP53, RB1, or PTEN pretreatment and after one cycle of abiraterone had a significantly shorter OS than patients without alterations at either time point [75]. Another study in mCRPC patients receiving ARTA therapy reported that genomic and transcriptional analysis of metastatic CRPC biopsies prior to enzalutamide treatment revealed that TP53 gene alterations as well as low AR transcriptional activity and activation of a stemness program were more common in enzalutamide-non-responders than responders [76].

In the SPARTAN trial assessing apalutamide plus ADT in high-risk non-metastatic CRPC patients, the frequency of TP53 alterations detected in ctDNA increased from baseline (22.2%) to end-of-study treatment (35.0%). In addition, in patients who received subsequent AR inhibitors, the presence of TP53 alterations was significantly associated with poor OS [59].

In mCRPC patients receiving docetaxel chemotherapy, cell-free DNA (cfDNA) alterations were analyzed before and after starting chemotheray, showing that cfDNA levels of TP53, RB1, and PTEN alterations remained relatively stable, which may indicate the peristence of clones associated with resistance to therapy [77]. Also, mCRPC patients with TP53 or RB1 defects had a significantly shorter PFS than those without these defects after docetaxel chemotherapy (4.8 vs. 8.0 months) [60].

In a retrospective study of mCRPC patients treated with Radium-223, TP53 alterations were present in 51.7% of patients, but no association of TP53 status between PSA response to Radium-223 and other clinical outcomes was detected [61].

For mCRPC patients treated with 177-Lutetium-PSMA, two single-center studies reported that alterations in TP53 were not associated with 177-Lu-PSMA outcomes [78,79], whereas a larger multi-center retrospective study and a study from a phase I/II trial on 177-Lu-PSMA therapy found an association between the presence of at least one mutation in TP53, RB1 or PTEN tumor suppressors with shorter PFS and OS [80,81].

A treatment option for mCRPC patients having received several lines of conventional systemic therapies is bipolar androgen therapy (BAT), whereby serum testosterone is cycled from supraphysiologic to near-castrate levels each month. Interestingly, in mCRPC patients who achieved deep responses to bipolar androgen therapy (>70% PSA reduction), pathogenic mutations in TP53 and/or a homologous recombination DNA repair gene were detected [34]. In particular, mCRPC patients that harbored pathogenic alterations in at least two of three genes (TP53, PTEN, and RB1) had significantly improved PFS and OS rates when receiving BAT [35].

## 4. *TP53* as a Therapeutic Target in Prostate Cancer

Due to its role as a transcription factor, WT p53 has long been considered undruggable, but emerging therapies seek to either restore WT function or exploit vulnerabilities associated with mutant p53. Therapeutic strategies for targeting p53 in cancer include restoring WT function in cancers with mutant p53 or inhibiting negative regulators like MDM2 and MDMX in cancers with WT p53 [24]. Mutant p53-targeted therapies aim to destabilize the mutant protein, which is usually highly expressed in cancer cells but not in normal cells. Moreover, research has demonstrated that even partial restoration of p53 activity can induce tumor regression, especially through effects on the tumor microenvironment [82]. Currently, clinical trials investigating these drugs are mostly phase I/II basket trials that recruit different patient populations with solid malignancies (i.e., NCT04585750, NCT06386146, NCT03975387, and NCT02264613), and few preclinical and clinical trials exist for PCa that evaluate p53 as a therapeutic target (Table 2). In 2011, the first in-human data of APR-256, a small molecule structural corrector that binds selectively to the p53 Y220C mutant protein and restores the p53 WT conformation, were published in refractory hematologic malignancies and prostate cancer [83]. In a phase Ib trial, eprenetapopt (APR-246) was tested again in combination with pembrolizumab in advanced or metastatic solid tumors. A total of 37 patients received the therapy within the study, only a few (<3) of them with prostate cancer, but the drug combination was well tolerated [84].

RG7112 binds MDM2 and inhibits its interaction with p53, thereby decreasing the negative effect of MDM2 on p53 stability. In a preclinical study, RG7112 led to cell cycle arrest and apoptosis in p53-WT cell models. The study group also tested the MDM2 antagonist in the androgen-dependent PCa cell line LNCaP and found a strong synergistic effect of androgen deprivation combined with RG7112 [85]. The drug was tested in a phase I trial in leukemia patients but has yet to be tested in PCa patients [86]. Another MDM2 antagonist, nutlin-3, has also been tested in the LNCaP cell line, showing promising preclinical results, but was again tested in p53 WT cells, therefore targeting p53 but not its mutated form [87]. In a different trial, nutlin-3 was tested as a radiosensitizing compound in three different PCa cell lines, including p53 WT and mutant cell lines. The results showed that nutlin-3 decreased the survival of cells after radiation independent of p53 [88].

Since aggregates formed by the mutant p53 can lead to loss of function of p53 tumor suppression and the gain of novel oncogenic functions, an inhibitor of p53 aggregation was tested in another preclinical study. ReACp53 was tested in p53-mutant PCa cell lines, and the results showed that the drug restored the transcriptional effects of p53 and reduced DNA synthesis of p53 mutant cells, thereby being another interesting potential target for further clinical trials [89]. In conclusion, reactivating p53 presents a promising therapeutic avenue, and further studies will show whether integration of p53-targeting strategies into clinical practice is possible.

**Table 2:** Clinical and preclinical trials investigating p53-associated targets in oncologic treatment strategies against PCa [83,84,85,87,88,89].

Drug	Target	Trial Design	Authors
APR-256	Restoration of transactivation of WT TP53 target genes	Phase I	S. Lehmann et al.; H. Park et al. [83,84]
RG7112	MDM2 inhibition	Preclinical	Christian Tovar et al. [85]
Nutlin-3	MDM2 antagonist	Preclinical	Stéphane Supiot et al.; Christian Tovar et al. [87,88]
ReACp53	Inhibition of p53 aggregation	Preclinical	Yaqun Zhang et al. [89]

## 5. Conclusions and Future Directions

This review underscores the role of *TP53* in the progression and treatment resistance of PCa. *TP53* mutations are frequent in advanced and metastatic stages, contributing to genomic instability, aggressive tumor behavior, and poor clinical outcomes. Mutations in *TP53* could serve as potential biomarkers for predicting disease progression and treatment resistance, particularly in castration-resistant disease. With the development of liquid biopsy strategies, analyzing circulating tumor cells (CTCs) or circulating tumor DNA (ctDNA), the presence of *TP53* mutations can be detected throughout the natural history of disease and may serve as a prognostic biomarker, guiding clinical decision-making regarding early treatment intensification strategies in patients with a potentially highly aggressive disease. Despite its prevalence, therapeutic strategies targeting *TP53* are still in the early stages, with preclinical and early-phase clinical trials investigating potential targets in oncologic therapeutic strategies. The development of novel approaches, including mutant-specific p53 inhibitors, MDM2 antagonists, and combination therapies, may provide future therapeutic options in managing *TP53*-altered PCa. Advancements in liquid biopsy and genomic profiling technologies can further enhance the detection of *TP53* mutations, enabling personalized treatment approaches. Future research should focus on evaluating the interaction between *TP53* alterations and other oncogenic drivers, possibly exploring synergistic effects and validating p53-targeting agents in large clinical trials.

## Figures and Tables

**Table 1:** Trials reporting the impact of TP53 mutations on clinical prognostic factors in different stages of disease [46,48,52,53,55,62,63,67,68].

Authors	Disease Setting	Impact of TP53 Mutations on Prognosis
Velho et al. [46]	High-risk localized PCa	Association with metastasis formation in univariate analysis
Nientiedt et al. [48]	Intermediate-/high-risk localized PCa	Independent risk factor for PSA failure or PSA persistence after radical prostatectomy
Alshalalfa et al. [52]	Localized PCa samples	TP53 alterations from localized PCa samples were associated with a higher risk of widespread metastasis (5 or more sites), higher risk of liver and bone metastasis, and shorter OS
Deek et al. [67]	Oligometastatic HSPC	Shorter rPFS and time to castration resistance
Sutera et al. [68]	Oligometastatic HSPC	Lower long-term control rate (no radiographic progression at last follow-up) and higher risk of bone failure in patients with TP53 mutations
Hamid et al. [52]	mHSPC	Shorter event-free survival and time to castration resistance and shorter OS in patients with TP53, PTEN, or RB1 mutations
Watson et al. [62]	Metastatic PCa	Shorter OS in patients with any TP53 alteration
Zhou et al. [53]	Metastatic PCa	Shorter OS in patients with TP53 mutations, especially in TP53 truncating mutations
Cussenot et al. [55]	Metastatic PCa	94% of samples collected in androgen deprivation-naïve patients; TP53 mutations associated with a higher risk of visceral metastasis and an early age at death
Hamid et al. [52]	mCRPC	Shorter OS (not clinically significant) in patients with TP53, PTEN, or RB1 mutations
